# Effects of Aerobic Exercise Training on Cardiac Renin-Angiotensin System in an Obese Zucker Rat Strain

**DOI:** 10.1371/journal.pone.0046114

**Published:** 2012-10-12

**Authors:** Diego Lopes Mendes Barretti, Flávio de Castro Magalhães, Tiago Fernandes, Everton Crivoi do Carmo, Kaleizu Teodoro Rosa, Maria Claudia Irigoyen, Carlos Eduardo Negrão, Edilamar Menezes Oliveira

**Affiliations:** 1 Laboratory of Biochemistry and Molecular Biology of Exercise, School of Physical Education and Sport, University of São Paulo, São Paulo, Brazil; 2 Experimental Physiopathology, University of São Paulo, Medical School, São Paulo, Brazil; 3 Heart Institute (InCor), University of São Paulo, Medical School, São Paulo, Brazil; University of Sao Paulo, Brazil

## Abstract

**Objective:**

Obesity and renin angiotensin system (RAS) hyperactivity are profoundly involved in cardiovascular diseases, however aerobic exercise training (EXT) can prevent obesity and cardiac RAS activation. The study hypothesis was to investigate whether obesity and its association with EXT alter the systemic and cardiac RAS components in an obese Zucker rat strain.

**Methods:**

The rats were divided into the following groups: Lean Zucker rats (LZR); lean Zucker rats plus EXT (LZR+EXT); obese Zucker rats (OZR) and obese Zucker rats plus EXT (OZR+EXT). EXT consisted of 10 weeks of 60-min swimming sessions, 5 days/week. At the end of the training protocol heart rate (HR), systolic blood pressure (SBP), cardiac hypertrophy (CH) and function, local and systemic components of RAS were evaluated. Also, systemic glucose, triglycerides, total cholesterol and its LDL and HDL fractions were measured.

**Results:**

The resting HR decreased (∼12%) for both LZR+EXT and OZR+EXT. However, only the LZR+EXT reached significance (p<0.05), while a tendency was found for OZR versus OZR+EXT (p = 0.07). In addition, exercise reduced (57%) triglycerides and (61%) LDL in the OZR+EXT. The systemic angiotensin I-converting enzyme (ACE) activity did not differ regardless of obesity and EXT, however, the OZR and OZR+EXT showed (66%) and (42%), respectively, less angiotensin II (Ang II) plasma concentration when compared with LZR. Furthermore, the results showed that EXT in the OZR prevented increase in CH, cardiac ACE activity, Ang II and AT2 receptor caused by obesity. In addition, exercise augmented cardiac ACE2 in both training groups.

**Conclusion:**

Despite the unchanged ACE and lower systemic Ang II levels in obesity, the cardiac RAS was increased in OZR and EXT in obese Zucker rats reduced some of the cardiac RAS components and prevented obesity-related CH. These results show that EXT prevented the heart RAS hyperactivity and cardiac maladaptive morphological alterations in obese Zucker rats.

## Introduction

According to the World Health Organization, over 1.7 billion people worldwide are overweight or obese [1]. Moreover, obesity is one of the major epidemics expanding in Western countries today, and increases the prevalence of most cardiovascular risk factors independently of other associated diseases. One of the most common independent cardiac features in obesity is cardiac hypertrophy (CH) [2]–[3]. The physiopathology of CH in obesity is complex and involves several factors such as hemodynamic, metabolic and neurohumoral factors [4].

The RAS is one of the most important neurohumoral contributors to the progression of pathological CH [5]. In the classical RAS pathway, angiotensinogen is cleaved by renin to produce angiotensin I (Ang I), which is converted to angiotensin II (Ang II) by angiotensin converting enzyme (ACE). Ang II is an important factor in cardiac remodeling, growth and apoptosis through the activation of specific receptors [5]. Nowadays, the importance of RAS located in specific tissues, such as heart and adipose tissue is well recognized, and its pharmacological inhibition has gained support [6]. Some authors have demonstrated that the use of ACE inhibitors can prevent obese cardiac cardiomyopathy, with improvements in cardiac metabolism and function, and reduction of CH in obese Zucker rats [7]–[8]. However, there is still controversy about the modulation of cardiac RAS in obesity, since some studies have observed an increase in some of the components of cardiac RAS, whereas others found no difference [9]–[11]. Furthermore, these results were demonstrated in independent studies, so the literature lacks studies demonstrating the association between each of the RAS components with obesity.

In addition, there is a growing body of evidence showing that aerobic exercise training (EXT) can reduce a number of cardiovascular risk factors [12]–[14]. Indeed, EXT has been recognized as an important non-pharmacological strategy to prevent obesity and related disorders. Furthermore, a recent study in our lab demonstrated that EXT induces effects on the RAS, such as decreased cardiac ACE and Ang II and increased cardiac ACE2 and Ang 1–7 [15] in healthy rats. In addition, Pereira et al [12] using a genetic mouse model also showed a beneficial effect of aerobic exercise on cardiac RAS components. However, to the best of our knowledge, the effect of EXT on cardiac RAS components in obese rats has not been studied yet.

Therefore, the present study was undertaken to test 2 hypotheses: 1) Whether the cardiac and systemic RAS components are changed in obese Zucker rats; and 2) Whether EXT alters the RAS components and prevents pathological CH in obese Zucker rats.

## Materials and Methods

### Experimental animals

Twenty male Zucker rats at 20 weeks of age were assigned to four groups (n = 5 each): Lean Zucker rat (LZR), lean Zucker rat plus exercise training (LZR+EXT), obese Zucker rat (OZR), obese Zucker rat plus exercise training (OZR+EXT). The animals were housed in standard cages and food and water were provided *ad libitum.* The environmental temperature was kept at 23±1°C and an inverted 12∶12 h dark-light cycle was maintained throughout the experiment.

All protocols and surgical procedures used were in accordance with the guidelines of the Brazilian College for Animal Experimentation and were approved by the Ethics Committee (1023/07) of the Institute of Biomedical Science of the University of Sao Paulo.

### Exercise protocol

Swimming training was performed as previously described [16]. Animals were trained in a swimming apparatus specially designed to allow individual exercise training of rats in warm water at 30–32°C. Physical training consisted of swimming sessions of 60-min duration, 5 days/wk, for 10 wk, wearing caudal dumbbells weighing 5% of their body weight. All animals were weighed once a week and the workload was adjusted to body weight variations. Sedentary groups were placed in the swimming apparatus for 10 minutes twice a week without workload. This protocol is defined as a low intensity, long training period, effective for promoting cardiovascular adaptations and increase in muscle oxidative capacity [16].

### Arterial heart rate and systolic blood pressure

Heart rate (HR) and systolic blood pressure (SBP) were determined non-invasively in the four experimental groups in the last week of the training protocol, using a computerized tail-cuff plethysmograph system (IITC Life Science Inc. CA USA). Rats were acclimatized to the apparatus during daily sessions over a period of 3 days.

### Echocardiography

Echocardiography features were obtained in accordance with the recommendations of the American Society of Echocardiography [17]. The animals were anesthetized intraperitoneally with a mixture of Xylazine (10 mg/kg) and ketamine (90 mg/kg). Transthoracic echocardiography was performed in the last week of the training protocol by only one observer using Sequoia 512 equipment (ACUSON Corporation, Mountain View, CA) and a 10 to 14 MHz multifrequency linear transducer. Images were obtained with the transducer placed on the animal's shaved chest (lateral recumbence). All measurements were performed by the same observer based on the average of 3 consecutive cardiac cycles. Left ventricle (LV) mass was calculated using the following formula, assuming a spherical LV geometry and validated for use in rats: LV mass = 1.047×[(LVd+PWd+IWd)3-LVd3], where 1.047 is the specific gravity of muscle, LVd is LV end-diastolic diameter and PWd and IWd are end-diastolic posterior and interseptum wall thicknesses, respectively. LV shortening was calculated as (LVd-LVs)/LVdx100, where LVs is LV end-systolic diameter. LV ejection fraction was calculated according to the Teichholz formula. Two-dimensionally guided pulsed Doppler recordings of LV transmitral flow were obtained from the apical 4-chamber view. E and A waves were measured by the peak velocity of the mitral valve. The E wave represents passive ventricular filling and the A wave represents active filling with atrial systole. Isovolumic relaxation time was taken as the time from aortic valve closure to the onset of mitral flow. The myocardial performance index was calculated by the sum of isovolumic contraction time and isovolumic relaxation time divided by the ejection time.

### Blood and Tissue Harvesting

Twenty four hours after the last training session, the rats were killed after twelve hours of fasting, by quick decapitation without prior anesthesia and blood and tissue samples were harvested. In addition, the LV, visceral fat pad (VFP) and tibia were harvested. The VFP and LV were weighted. The tibia lengths were determined and the hypertrophy index of each animal (weight of LV in g/tibia length in cm) was calculated. The tissues, serum and plasma were frozen at −80°C and used within 1 month for enzyme assays and mRNA and protein preparations.

### Serum glucose, cholesterol, HDL, LDL and triglycerides

Serum glucose, cholesterol, HDL, LDL and triglyceride levels were measured by spectrophotometer according to the procedures described in commercially available kits (Labtest).

### Angiotensin-converting enzyme activity assay

ACE activity in rat serum and cardiac tissue (LV) extracts were determined using Abz-FRK(DNP)P-OH derivatives as substrates by continuously measuring the fluorescence according to Alves et al [18]. Tissue samples were quickly harvested, homogenized in Tris-HCl buffer, pH 7.0, containing 50 mM NaCl and centrifuged at 1000 g for 10 min. The assays were performed at 37°C in 0.1 M Tris-HCl buffer, pH 7.0, containing 50 mM NaCl and 10 µM ZnCl_2_. The hydrolysis rate of intramolecularly quenched fluorogenic substrate Abz-YRK-(Dnp)p (10 µM) incubated with aliquots of tissue homogenate and serum for 30 min at 37°C was assessed to obtain ACE enzymatic activity. Fluorescence increments throughout the period of time were read at 420 nm emission: 320 nm excitation. Tissue and serum ACE activity were expressed as UF.min^−1^.mg^−1^of protein ×1000. The protein content was determined by the Bradford methods [19] using bovine serum albumin as the standard (Bio-Rad Protein Assay).

### Cardiac angiotensin-converting enzyme 2 (ACE2) activity

ACE2 activity was determined in LV tissue by the same method described above. However, this method uses a fluorescent peptide Abz-APK(Dnp)-OH in 0.2 M Tris-HCl buffer, 200 mM NaCl, 2 µg BSA, pH 7.5, which is hydrolyzed by ACE2 (Kcat/Km = 3,5×10^4^ M^−1^.s^−1^). ACE2 activity was expressed in UF.min^−1^.mg^−1^of protein.

### Measurement of Angiotensin II

Plasma and LV Ang II levels were determined by ELISA, according to the manufacturer's instructions (SPI-BIO). To determine plasma Ang II concentration the first 3 ml of trunk blood was rapidly collected in chilled glass tubes containing a mixture of protease inhibitors [potassium EDTA (25 mmol), *ο*-phenanthroline (0.44 mmol), pepstatin A (0.12 mmol), and 4-(chloromercuribenzoic acid) (1 mmol)] to prevent the *in vitro* production and degradation of angiotensin peptides. The blood was centrifuged, and plasma was separated and stored at −20°C. LV was homogenized in lyses buffer (sodium phosphate 0.1 M, sucrose 0.34 M, NaCl 0.3 M) containing a mixture of protease inhibitors and centrifuged at 10 000×g, 4°C, for 10 min., to determine the measurement of cardiac Ang II. The supernatant and plasma were collected and passed through phenyl silica cartridges (Sep-Pak C18 columns, Waters), and the absorbed angiotensin was eluted with methanol. Eluate was dried in a vacuum centrifuge and the pellet was re-suspended in EIA buffer, vortexed and centrifuged at 3000 g for 10 minutes at 4°C. The results were expressed pg/ml. The tissue protein content was determined by the Bradford methods [19] by using bovine serum albumin as the standard (Bio-Rad Protein Assay).

### Western blot analysis

The frozen ventricles were thawed and minced into small pieces and homogenized in cell lysis buffer containing 100 mM Tris, 50 mM NaCl, 10 mM EDTA, 1% TritonX-100 and a mixture of protease inhibitors. Insoluble heart tissues were removed by centrifugation at 3,000×g, 4°C, for 10 min. Samples were loaded and subjected to SDS-PAGE in 10% polyacrylamide gels. After electrophoresis, proteins were electro-transferred to nitrocellulose membrane (Amersham Biosciences; Piscataway, NJ). Equal loading of samples (60 µg) and even transfer efficiency were monitored with the use of 0.5% Ponceau S staining of the blot membrane. The blot membrane was then incubated in a blocking buffer (5% nonfat dry milk, 10 mM Tris-HCl, pH 7.6, 150 mM NaCl, and 0.1% Tween 20) for 2 h at room temperature and then probed with a polyclonal antibody directed against AT1 or AT2 (1∶1000 or 1∶1000, respectively; Santa Cruz Biotechnology Inc., Santa Cruz, CA, USA) at room temperature. Primary antibody binding was detected with the use of peroxidase-conjugated secondary antibodies, and enhanced chemiluminescence reagents (Amersham Biosciences; Piscataway, NJ) were used to visualize the autoradiogram, which was later exposed to photographic film. The film was developed and the bands were analyzed using Scion Image software (Scion Corporation based on NIH image). GAPDH expression levels were used to normalize the results.

### Reverse Transcriptase/Real-Time Polymerase Chain Reaction

The relative gene expression of α myosin heavy chain (αMHC) and β myosin heavy chain (βMHC) were analyzed by Real-Time Polymerase Chain Reaction PCR (RT-PCR). Total RNA was isolated from LV with Trizol reagent (GIBCO Invitrogen). Total RNA concentration and integrity were assessed and RT-PCR was performed. RNA samples were quantified by absorbance at 260 nm and 280 nm in order to determine their concentration and purity levels. Only samples with 260 nm/280 nm index between 1.8–2.2 were considered. The αMHC and βMHC mRNA expression were assessed by oligonucleotide primers as follows: for αMHC, (5′-CGA GTC CCA GGT CAA CAA G-3′) and (5′-AGG CTC TTT CTG CTG GAC C-3′); for βMHC, (5′-CAT CCC CAA TGA GAC GAA G-3′), and (5′-AGG CTC TTT CTG CTG GAC A-3′) and for ACE (5′CAG GAA CGT GGA ACT TGG A 3′) and (5′CTT TGA CGC AAG CAT CAC C 3′). The expression of cyclophilin A (5′-AAT GCT GGA CCA AAC ACA AA-3′) and (5′-CCT TCT TTC ACC TTC CCA AA-3′) was measured as an internal control for sample variation in RT reaction. Real-Time PCR amplifications were performed with an *ABI Prism 7700 Sequence Detection System* (Applied Biosystems), using *SYBR Green PCR Master Mix* (Applied Biosystems). All samples were assayed in triplicate. The results were quantified as Ct values, where Ct is defined as the threshold cycle of the polymerase chain reaction at which the amplified product is first detected. Values for the control gene (cyclophilin) were used to standardize the results in order to compensate for differences in RNA content among the samples. The comparative threshold (C_T_) cycle method was used for data analyses. C_T_ indicates the fractional cycle number at which the amount of amplified target reaches a fixed threshold, and ΔC_T_ is the difference in threshold cycle for target (αMHC, βMHC, ACE) and control (cyclophilin). The levels of (αMHC, βMHC, ACE) gene expression were given by 2^−ΔΔCT^; where ΔΔC_T_ is the ΔC_T_ value subtracted from ΔC_T_ of lean untrained rats. Finally the 2 fold ΔΔC_T_ was calculated.

### Statistical Analysis

All the data were subjected to statistical analyses using the SAS program. A mixed model was used, with body composition (BC) (lean and obese) and exercise training (EXT) (untrained and trained) as fixed factors for all variables, except for body weight, for which a time factor was included. A Delta (Δ) Body weight (Final minus initial protocol body weight) was calculated. When significance F value was obtained a post-hoc test with Tukey's adjustment was performed for multiple comparison purposes. Results were expressed as means ± SEMs. Statistical significance was set at P<0.05.

## Results

### Body weight, visceral fat pad and left ventricle ratio

Table 1 shows the effects of time, body composition (BC) and EXT on the Body Weight (BW) and the effects of BC and EXT on the visceral fat pad (VFP) and left ventricle (LV) ratio. Both obese groups (OZR, OZR+EXT) showed higher BW before and after the training protocol when compared with the Lean groups (LZR, LZR+EXT) (p<0.0001), while no significant EXT effect was observed. However, when Δ BW was calculated, a significant difference was observed (p<0.05) between untrained OZR and the other groups (LZR, LZR+EXT and OZR+EXT). As regards the LV ratio, there was a significant BC effect (p<0.0001) and EXT effect (p<001). Post hoc analyses for individual conditions showed that untrained OZR had a significant increase (65%) in LV ratio in comparison with untrained and trained LZR (p<0.001), however the OZR+EXT showed a significant reduction (15%) in comparison with untrained OZR (p<0.05). The VFP showed similar effect results to those of the LV ratio. However, the differences were bigger (OZR increased 750% in comparison with LZR) and the exercise training in OZR+EXT decreased (20%) VFP in comparison with untrained OZR. In addition, these latter results showed a correlation between the gain in VFP and LV ratio (r^2^ = 0.73; p<0.0001).

**Table 1:** Effects of body composition and aerobic exercise training on BW, HWR, VFP, HR and SBP.

Variables	Groups				P value		
	LZR	LZR+EXT	OZR	OZR+EXT	BC	EXT	Interaction
BW before, g	349±10.7	342±12.3	507±17.5** &&	532±12.6** &&	<0.0001	NS	NS
BW after, g	378±13.4	383±12.5	600±17.1** &&	580±18.4** &&	<0.0001	NS	NS
Delta BW	45±5.7	40±4.5	93±13	48±10##	NS	NS	<0.01
LVR, g/cm	1.94±0.1	1.8±0.1	2.65±0.1* &	2.31±0.1* & #	<0.0001	<0.001	NS
VFP, g	10.76±0.97	9.92±1.30	85.00±6.06** &	68.25±2.36** & #	<0.0001	<0.001	NS
HR, bpm	436±15.3	382±9.5* #	433±21.6	383±5.1*	NS	<0.01	NS
SBP, mm/Hg	115±2	116±1.1	124±1.7* &	127±3.8* &	<0.001	NS	NS

LZR. lean Zucker rat; OZR. obese Zucker rat; LZR+EXT. lean Zucker rat plus exercise training; OZR+EXT. obese Zucker rat plus exercise training. (BW) body weight; (LVR) Left ventricle ratio; (VFP) visceral fat pad; (HR) heart rate; (SBP) systolic blood pressure. Data are reported as means ± SD of 5 animals in each group.

* p<0.05 versus lean untrained,

** p<0.001 versus lean untrained,

& p<0.05 versus lean trained,

&& p<0.001 versus lean trained,

# p<0.05 versus obese untrained.

### Hemodynamic parameters

In order to verify the hemodynamic changes and cardiovascular adaptations in BC and EXT, the HR and SBP were measured at the end of the training protocol as shown in Table 1. There was a significant EXT effect (p<0.01) for HR and a significant BC effect (p<0.001) for SBP. When comparing untrained LZR versus LZR+EXT, the post hoc analyses showed significant decreased (12%) in HR (p<0.05), while only a marginal significance was found for OZR versus OZR+EXT (p = 0.07). The absence of statistical significance of the HR in the OZR+EXT could be due to the lower sample size in the study (N = 5) per group.

### Cardiac parameters

Table 2 shows the results obtained by echocardiography at the end of the EXT protocol. The untrained OZR had (26%) LV mass increase in comparison with both lean groups (LZR and LZR+EXT) (p<0.05), while the OZR+EXT had (17%) LV mass decrease in comparison with OZR (p<0.05) as indicated by individual comparisons in the post hoc test. These results confirmed our previous data obtained by means of the LV ratio. Cardiac functions measured by systolic and diastolic parameters showed no statistical differences, however, although there was no statistical difference (p = 0.07) a (25%) reduced time E/A wave ratio was found when OZR was compared with untrained LZR, while OZR+EXT had values close to those of the lean control group.

**Table 2:** Effects of body composition and aerobic exercise training on cardiac function.

Variables	Groups				P value		
	LZR	LZR+EXT	OZR	OZR+EXT	BC	EXT	Interaction
Systolic function							
Ef (%)	0.83±0.03	0.78±0.08	0.88±0.02	0.84±0.03	NS	NS	NS
FS (%)	0.45±0.02	0.42±0.08	0.52±0.03	0.48±0.03	NS	NS	NS
Diastolic function							
E/A wave ratio (m/s)	2.04±0,11	2.14±0.26	1.54±0.26	2.20±0.62	NS	NS	NS
IVRT (ms)	22.00±2.45	29.40±9.13	28.00±4.64	29.50±3.46	NS	NS	NS
LVM (g)	0.98±0.04	1.17±0.03*	1.24±0.07*	1.02±0.12# &	<0.05	NS	<0.0001

LZR, lean Zucker rat; OZR, obese Zucker rat; LZR+EXT, lean Zucker rat plus exercise training; OZR+EXT, obese Zucker rat plus exercise training. (Ef) Ejection fraction; (FS) fractional shortening; (IVRT) isovolumetric relaxation time; left (LVM) ventricle mass. Data are reported as means ± SD of 5 animals in each group.

* p<0.05 versus lean untrained,

& p<0.05 versus lean trained,

# p<0.05 versus obese untrained.

### Metabolic parameters

To verify whether this obesity model leads to other metabolic risk factors, and whether the EXT alters these parameters after the treatment protocol, some plasma components, such as glucose, total cholesterol, high-density lipoprotein-cholesterol (HDL), low-density lipoprotein-cholesterol (LDL) and triglycerides were analyzed. The results are shown in Table 3. In this model, glucose remained the same in all groups irrespective of BC and EXT. Triglycerides, total cholesterol and LDL were higher in the both obese groups (p<0.0001), when compared with both lean groups. On the other hand only untrained OZR showed decreased HDL compared to both lean groups. In addition, OZR+EXT showed lower triglycerides (p<0.001) and LDL cholesterol (p<0.01) levels in comparison with those of the OZR.

**Table 3:** Effects of body composition and aerobic exercise training on metabolic parameters.

Variables	Groups				P value		
	LZR	LZR+EXT	OZR	OZR+EXT	BC	EXT	Interaction
Glucose(mg/dl)	122±6	123±5	133±9	141±7	NS	NS	NS
Triglycerides(mg/dl)	39±3	34±4	235±7** &&	135±25** && ##	<0.0001	<0.001	<0.01
Cholesterol(mg/dl)	63±3	65±5	128±7*** &&&	129±9*** &&&	<0.0001	NS	NS
HDL(mg/dl)	43±3	44±9	26±4** &&	37±2	<0.01	NS	NS
LDL(mg/dl)	12±2	14±3	74±4*** &&&	45±7** && ##	<0.0001	<0.05	<0.01

LZR, lean Zucker rat; OZR, obese Zucker rat; LZR+EXT, lean Zucker rat plus exercise training; OZR+EXT, obese Zucker rat plus exercise training. (HDL) high density lipoprotein; (LDL) low density lipoprotein. Data are reported as means ± SD of 5 animals in each group.

** p<0.001 versus lean untrained,

*** p<0.0001 versus lean untrained,

&& p<0.001 versus lean trained,

&&& p<0.0001 versus lean trained

# p<0.05 versus obese untrained,

## p<0.001 versus obese untrained.

### α and β – Myosin Heavy Chain (MHC)

**Figure 1:**
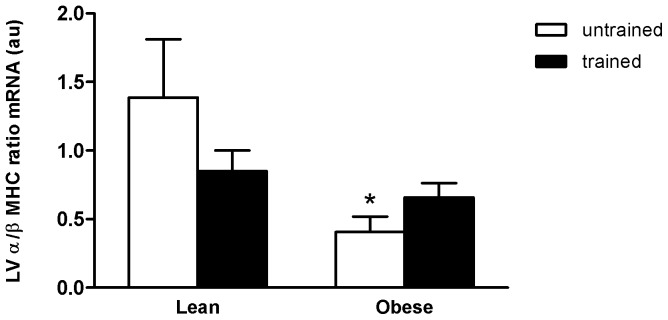
Effect of obesity and exercise training on α/β-MHC (alpha/beta-Myosin Heavy Chain) ratio in rat ventricles. Data are reported as means ± SEMs of 5 animals in each group. *p<0.05 versus lean untrained.

To gain better understanding of whether obese CH is physiological or pathological, pathological hypertrophy markers, α and β-MHC were analyzed. There was a significant BC effect on these cardiac markers (p<0.05), with (65%) decrease in untrained OZR shown in the post hoc test in comparison with the untrained LZR. On the other hand, although the α/β-MHC ratio in OZR+EXT group was not different from OZR, the EXT prevented the decrease in the α/β-MHC ratio, once the OZR+EXT were not different from the lean rats (Fig. 1).

### Systemic Renin-Angiotensin-System

**Figure 2:**
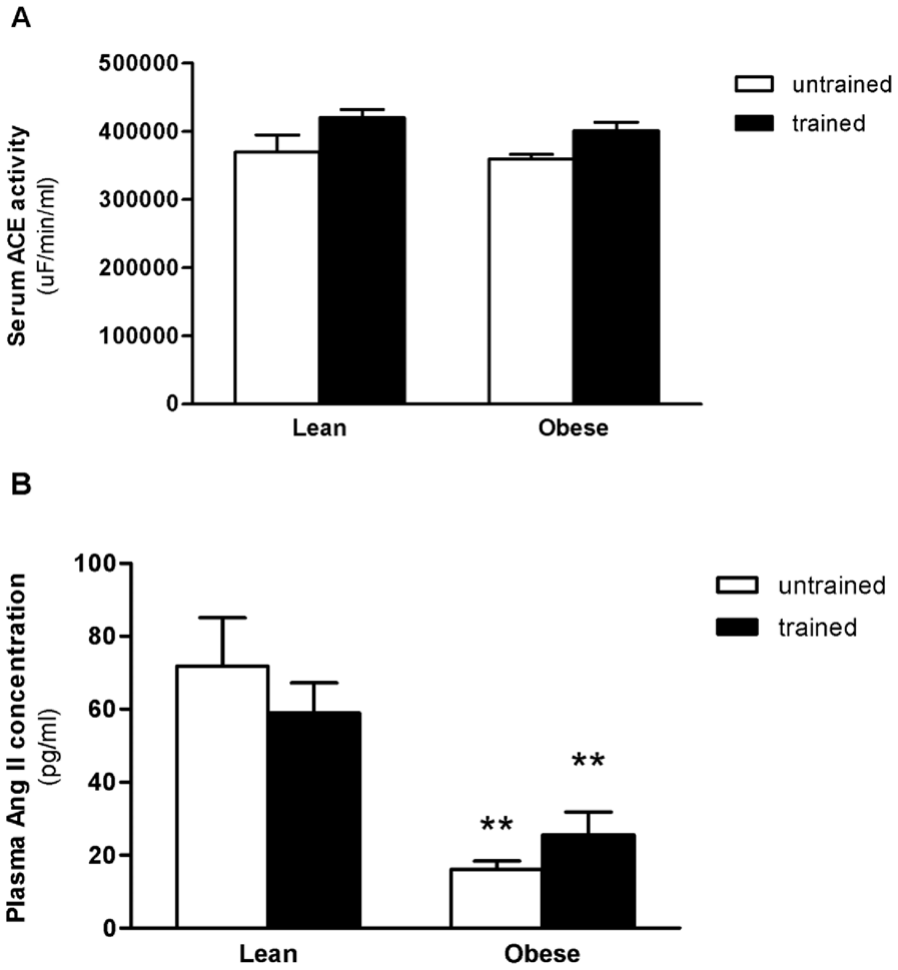
Systemic Renin-Angiotensin-System. A) Effect of obesity and exercise training on ACE activity. B) Effect of obesity and exercise training on Angiotensin II peptide concentration. Data are reported as means ± SEMs of 5 animals in each group. **p<0.001 versus lean untrained, ^&&^p<0.001 versus lean trained.

In order to investigate the role of RAS components in BC and EXT even further, the systemic RAS and cardiac components were evaluated. There were no significant effects of BC, EXT and interaction on serum ACE activity (Fig. 2a). However, as regards plasma Ang II concentration, untrained OZR showed a decrease (66%) compared with untrained LZR; and OZR+EXT a decrease (42%) compared with untrained LZR (Fig. 2b).

### Cardiac Renin-Angiotensin-System

**Figure 3:**
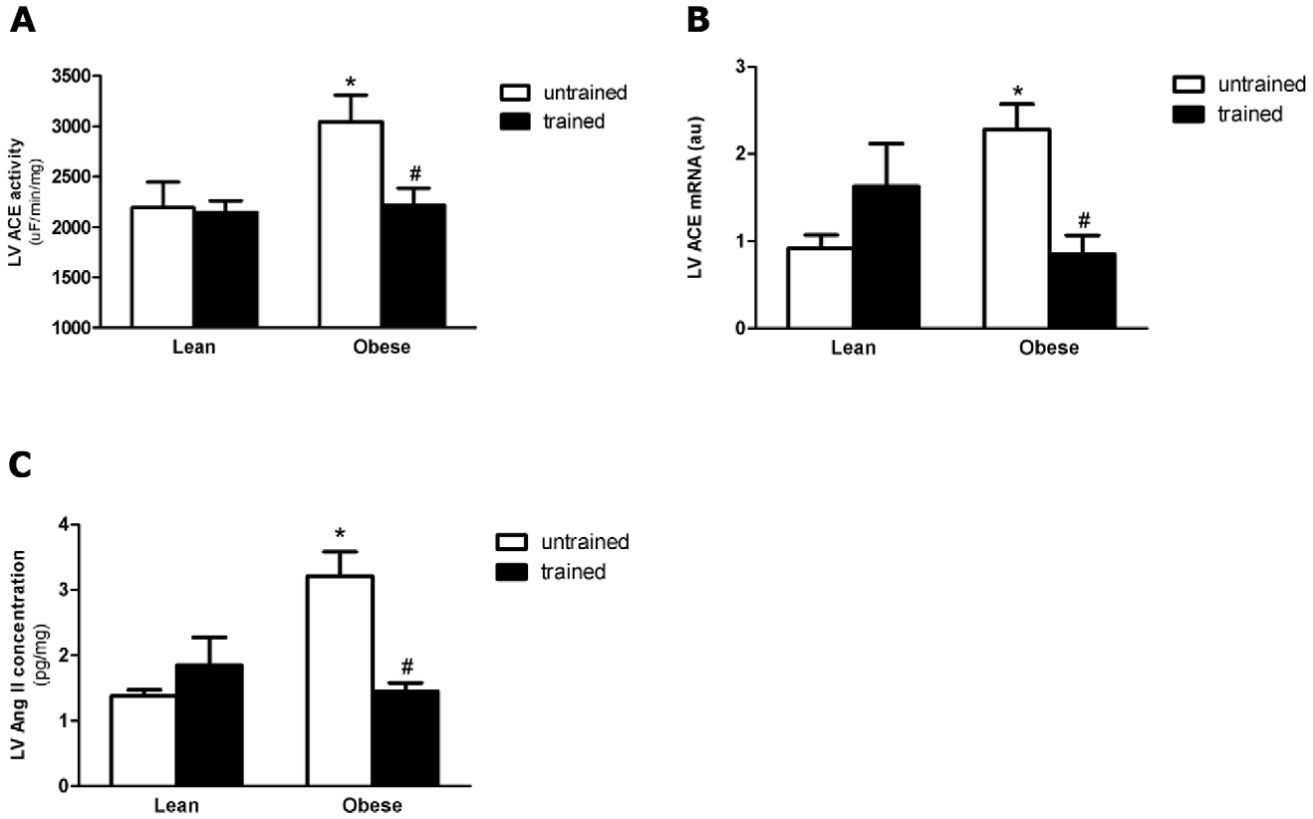
Cardiac Renin-Angiotensin-System. A) Effect of obesity and exercise training on Cardiac ACE activity. B) Effect of obesity and exercise training on ACE mRNA expression (Quantification of mRNA ACE was normalized against Cyclophilin mRNA). C) Effect of obesity and exercise training on concentration of Angiotensin II peptide in rat left ventricle. Data are reported as means ± SEMs of 5 animals in each group. *p<0.05 versus lean untrained, **p<0.001, ^&^p<0.001 versus lean trained, ^#^p<0.05 versus obese untrained.

**Figure 4:**
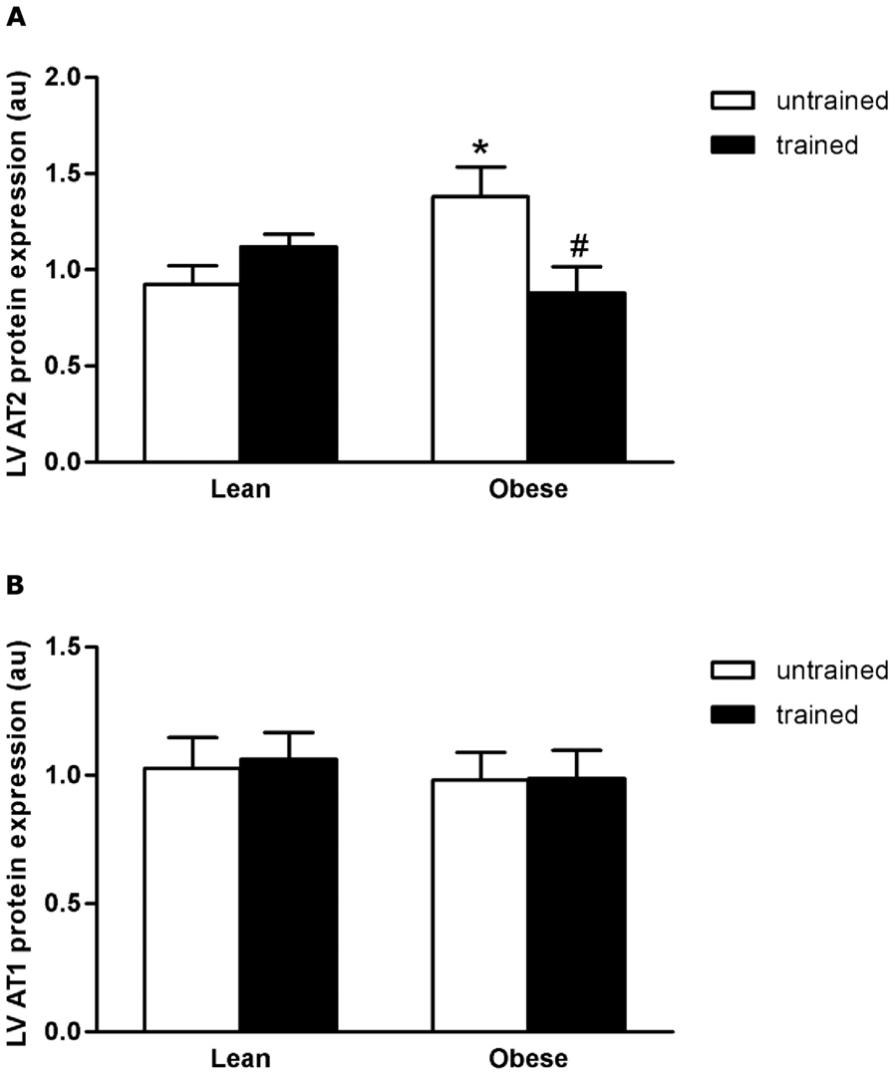
Cardiac Angiotensin II receptors. A) Effect of obesity and exercise training on AT1 protein expression analyzed by Western blot. B) Effect of obesity and exercise training on AT2 protein expression analyzed by western blot. Data are reported as means ± SEMs of 5 animals in each group. *p<0.05 versus lean untrained. ^#^p<0.05 versus obese untrained.

**Figure 5:**
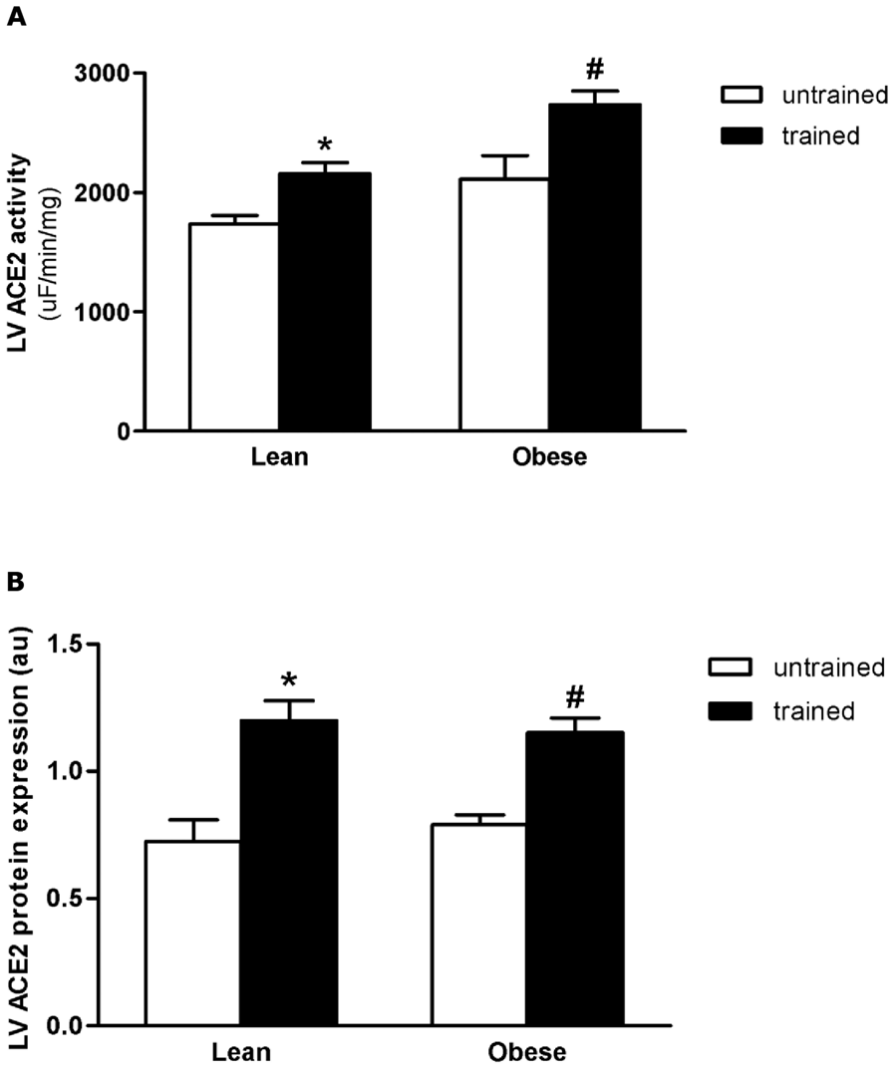
Cardiac Angiotensin Converting Enzyme 2 (ACE2). A) Effect of obesity and exercise training on Cardiac ACE 2. B) Effect of obesity and exercise training on ACE2 protein expression analyzed by western blot. Data are reported as means ± SEMs of 5 animals in each group. *p<0.05 versus lean untrained, ^#^p<0,05 versus obese untrained.

LV ACE activity and gene expression, ACE2 activity and protein expression, Ang II concentration and Ang II receptor (AT1 and AT2) protein expressions were measured in order to verify the relationship between cardiac alterations and local RAS in BC and EXT. Differently from the systemic RAS, there was significant increase (36% p<0.05; 148% p<0.05) in LV ACE activity and gene expression in untrained OZR, when compared with untrained LZR (Figures 3a and 3b). These alterations were accompanied by high levels of cardiac Ang II (untrained OZR showed an increase of 90% when compared with untrained LZR; p<0.001) and AT2 receptor (untrained OZR showed a 50% increase when compared with untrained LZR; p<0.05), (Figures 4 and 5a, respectively). AT1 protein expressions were the same for all groups. In the OZR+EXT, swimming training decreased ACE activity (27% decrease compared with OZR; p<0.05) and expression (63% decrease compared with OZR; p<0.05), Ang II concentration (44% decrease compared with OZR; p<0.05) and AT2 expression (35% decrease compared with OZR; p<0.05), (Figures 3a, 3b, 3c and 4a, respectively) being comparable with values shown for LZR. Moreover, EXT increased ACE2 activity (LZR+EXT showed a 24% increase compared with LZR; p<0.05 and OZR+EXT showed a 30% increase compared with OZR; p<0.05), and protein expression (LZR+EXT showed a 74% increase compared with OZR; p<0.05 and OZR+EXT showed a 81% increase compared with OZR; p<0.05) in both trained groups irrespective of BC, demonstrating the beneficial effect of exercise per se on ACE2, (Figures 5a and 5b).

## Discussion

Obesity is a chronic disease and can lead to several other pathologies, involving diabetes mellitus type 2, hypertension, some lipid disorders and other cardiac disorders [4], [20]–[21]. Indeed, obesity is an independent factor for the development of cardiac disorders, and this association is one of the most common causes of death [22]. Therefore, it is important to understand the mechanisms responsible for this pathological condition and create therapeutic strategies, such as the use of drugs or exercises that modulate obesity-related cardiac disease.

As shown in the literature, the genetically obese Zucker rat, a widely used model, has a large amount of adipose tissue and abnormally large numbers of large fat cells, which synthesize fatty acids and triglycerides at increased rates, which in turn, lead to some metabolic disorders involving triglyceride and cholesterol metabolism [23]–[25]. The obese group in the present study had elevated triglycerides, total cholesterol and LDL, and decreased HDL. On the other hand, swimming training in obese rats improved this condition by lowering triglycerides and LDL cholesterol and augmenting HDL cholesterol. These changes are important, because these metabolic alterations can contribute to cardiac modifications [26], such as cardiac metabolic disorders and increased cardiac mass [27], as seen in the Zucker model [8], [10]. Likewise, in the untrained obese Zucker rats in this study, increased cardiac mass, measured by cardiac weight and echocardiography were found. However, swimming training in the obese group decreased cardiac mass, which together with the metabolic parameters are important to improve cardiac conditioning in obesity. The higher cardiac mass in the untrained obese rats was accompanied by lower levels of molecular α/β MHC ratio. This modification suggests a cardiac pathological condition resembling fetal gene reprogram [28]. The molecular mechanisms involved in cardiac modifications in obesity and exercise training are not fully understood.

Studies that used ACE inhibitors have suggested that the RAS seems to play a critical role in cardiac modifications [7], [27]. However, although some studies have shown that the RAS can participate in cardiac diseases related to obesity, they did not measure the cardiac RAS components [8], [27]. For the first time, in the present study, the local cardiac RAS components were measured in obese rats, and here they provide direct evidence of involvement of the local cardiac RAS components in obese rats; and that local cardiac RAS involvement is independent of the systemic RAS in this obese model and that EXT can independently modulate local and systemic RAS. Furthermore, some authors have demonstrated that EXT can be an interesting tool for non-pharmacological treatment of some cardiovascular diseases [12]–[14], [29]. In this study it was demonstrated that EXT is an important therapeutic strategy to combat obesity by reducing weight gain, improving the metabolic parameters, and most importantly, down-regulating the pathological cardiac RAS components and bringing them to levels similar to those observed in LZR.

The local cardiac RAS changes were independent of circulating RAS, as lower levels of systemic Ang II were found for both obese groups with no change in serum ACE activity, it is not known by which means the systemic Ang II concentration is decreased despite normal ACE activity. Studies with focus on circulating RAS are controversial. Differences in obesity models seem to play a critical role in systemic RAS. Some studies with diet-induced obesity in Wistar and Sprague-Dawley rats showed increase circulating levels of Ang II [11], [30]. The systemic ACE was not measured in these studies. On the other hand, Engeli et al [31]. did not find any difference in the systemic Ang II levels in obese humans, while the renin, ACE and angiotensinogen levels were elevated. Up to now, there are no studies that measure plasma Ang II concentration in Zucker rats. Nevertheless, differently from other obesity models, the obese Zucker rats have low plasma levels of renin [32]–[34]. The lower renin levels could be the possible mechanism by which the Ang II was lower in our obese Zucker rats because, under most physiological conditions, the rate-limiting step for ANG II generation is the cleavage of Ang I from angiotensinogen by aspartyl protease renin. Furthermore, studies that found an increase in vascular Ang II sensitivity in obese Zucker rats [34]–[35], support our findings, since the lower circulating Ang II levels are probably responsible for this alteration in vascular sensitivity.

Differently from the systemic condition, in this obesity model an increase in cardiac ACE activity and gene expression was found, followed by higher levels of Ang II and AT2 receptor. On the other hand, reduced cardiac ACE, Ang II and AT2 receptor were observed in OZR+EXT, and these modifications were accompanied by a reduction in cardiac mass. Clinical and genetic studies have demonstrated an influence of RAS on CH [36]–[37]. Huang et al. [10] observed an increase in cardiac AT1 expression in Zucker rats and demonstrated that a decrease in cardiac mass is at least in part, associated with modulation of cardiac AT1 expression. However, in this study the Zucker rats were diabetics, which differs from our Zucker model. In the present study, it was not possible find an alteration in AT1 receptor protein expression, however, higher levels of AT2 receptor protein expression were observed in untrained obese rats. Unfortunately, there are no studies that have measured cardiac AT2 in obese rats. However, D'Amore et al. showed that in cardiomyocytes, the AT2 receptor causes constitutive growth and does not oppose the actions of the AT1 receptor. Another possible mechanism by which the AT2 expression is up-regulated, is to oppose some deleterious effects [38]–[39]. It is well established that the obese Zucker rat has cardiac metabolic disorders, which in turn lead to an increase in the apoptotic pathway [40]. Furthermore, as shown by Goldenberg et al, the increase in the AT2 leads to an increase in the apoptotic pathway [41]. Therefore, the increase in cardiac AT2 receptor protein levels could probably counteract the deleterious effect in this model, since exercise training in OZR+EXT decreased AT2 to normal levels when compared with their untrained littermates. Taken together, these results suggest that the increase in AT2 protein levels in the present study could have been produced by three main mechanisms: counteracting the deleterious effects of obesity, promoting cardiac hypertrophy and increasing apoptotic signaling.

The results of the present study also demonstrated that EXT down-regulated ACE, Ang II and AT2 in the obese group. These latter results are in agreement with previous results observed in the authors' laboratory, in which it was observed that aerobic exercise training in healthy Wistar rats had some effects on the RAS, such as decreased cardiac ACE and Ang II and increased cardiac ACE2 and Ang 1–7 [15]. Moreover, Pereira et al [12] in a genetic mouse model also showed a beneficial effect of aerobic exercise on the cardiac RAS components. Filho et al [29] showed that aerobic training increased LV Ang 1–7 levels in swimming-trained a model of spontaneously hypertensive rats. In parallel, it was observed that exercise increased both receptor Mass mRNA and protein levels in the heart, suggesting that an overall activation of the Ang 1–7 – Mass axis may be involved in the beneficial effects of physical training in hypertensive disease. Thus, it seems that exercise training alters the cardiac RAS components, and these modifications are beneficial to the heart in some cases of cardiac pathology.

Finally, irrespective of whether the animals were obese or not, exercise increased cardiac ACE2 in both trained groups, as shown in Figure 5. It is known that ACE2 can cleave Ang II into Angiotensin 1–7 (Ang 1–7) [42]. Ang 1–7 has a vasodilator effect and inhibits pathological cardiac growth [43]. Therefore, elevating ACE2 levels could be beneficial in obesity.

In summary, the results of the present study showed that cardiac mass, cardiac ACE activity and gene expression, as well as Ang II concentration were increased, although systemic Ang II was decreased in a Zucker genetic model of obesity. Exercise training increases cardiac ACE2 activity and protein expression and prevents the increase in cardiac mass, cardiac ACE, Ang II, AT2 caused by obesity. Therefore, the above results demonstrated that in a genetic model of obesity, pathological cardiac hypertrophy markers are increased together with increased cardiac RAS activity and expression; however, aerobic exercise training prevents cardiac hypertrophy in this model and decreases cardiac RAS activity and expression, suggesting that aerobic exercise training modulates cardiac RAS in the context of obesity.
